# Chromosomal Mapping of Repetitive DNAs in the Grasshopper *Abracris flavolineata* Reveal Possible Ancestry of the B Chromosome and H3 Histone Spreading

**DOI:** 10.1371/journal.pone.0066532

**Published:** 2013-06-27

**Authors:** Danilo Bueno, Octavio Manuel Palacios-Gimenez, Diogo Cavalcanti Cabral-de-Mello

**Affiliations:** Universidade Estadual Paulista (UNESP), Instituto de Biociências/IB, Departamento de Biologia, Rio Claro, São Paulo, Brazil; Wellcome Trust Centre for Stem Cell Research, United Kingdom

## Abstract

Supernumerary chromosomes (B chromosomes) occur in approximately 15% of eukaryote species. Although these chromosomes have been extensively studied, knowledge concerning their specific molecular composition is lacking in most cases. The accumulation of repetitive DNAs is one remarkable characteristic of B chromosomes, and the occurrence of distinct types of multigene families, satellite DNAs and some transposable elements have been reported. Here, we describe the organization of repetitive DNAs in the A complement and B chromosome system in the grasshopper species *Abracris flavolineata* using classical cytogenetic techniques and FISH analysis using probes for five multigene families, telomeric repeats and repetitive *C_0_t*-1 DNA fractions. The 18S rRNA and H3 histone multigene families are highly variable and well distributed in *A. flavolineata* chromosomes, which contrasts with the conservation of U snRNA genes and less variable distribution of 5S rDNA sequences. The H3 histone gene was an extensively distributed with clusters occurring in all chromosomes. Repetitive DNAs were concentrated in C-positive regions, including the pericentromeric region and small chromosomal arms, with some occurrence in C-negative regions, but abundance was low in the B chromosome. Finally, the first demonstration of the U2 snRNA gene in B chromosomes in *A. flavolineata* may shed light on its possible origin. These results provide new information regarding chromosomal variability for repetitive DNAs in grasshoppers and the specific molecular composition of B chromosomes.

## Introduction

Repetitive DNAs comprise a large portion of eukaryotic genomes, including tandem arrays and scattered repeats. Tandem repeats are represented by microsatellite, minisatellite, and satellite DNAs as well as some multigene families, while dispersed repeats are comprised of transposons and retrotransposons [1]–[3]. Among the multigene families, the ribosomal DNAs (rDNAs), followed by histone genes and to a lesser extent U small nuclear RNA (snRNA) genes, have been mapped cytogenetically, revealing clusters located in one chromosomal loci or dispersed in some chromosomes (see for example [4]–[11]). The spreading of these sequences has been attributed to transposition and ectopic recombination, as well as the possible involvement of extra chromosomal circular DNAs (eccDNA), which have been detected in *Drosophila*, *Arabdopsis thaliana* and human (see for example [4], [6]–[8], [12]–[19]).

In grasshoppers, the mapping of multigene families for rDNAs and histone genes has identified distinct patterns of chromosomal distributions. In particular the H3/H4 histone clusters are highly conserved in one chromosomal pair, and the rDNAs are variable due to amplification and dispersion of the clusters in some chromosomes. In some cases these sequences are co-located in the same chromosomal area [4], [5], [8]. Moreover the presence of multigene families in B chromosomes has been reported in some species (see references below).

The B chromosomes, also known as accessory or supernumerary elements, are dispensable chromosomes not required for normal organismal development, constituting a type of selfish DNA element [20], [21]. Since their discovery by Wilson [22], distinct B chromosomes have been described in all eukaryotic groups and occur in approximately 15% of species cytogenetically investigated [20]. Primary characteristics of B chromosomes include their remarkable accumulation due to irregular modes of inheritance; pairing incapacity during meiosis with standard A chromosomes; and accumulation of distinct repetitive DNAs, leading to species-specific evolutionary fates [20], [21], [23]–[25].

Concerning molecular composition of animal B chromosomes, among repetitive sequences the presence of satellite repeats, transposable elements and multigene families, mainly 45S rDNA, have been described [20]. In grasshoppers, the presence of satellite DNA and multigene families such 45S and 5S rDNA and H3/H4 histone genes have been described in distinct species such as *Dichroplus pratensis* [26], *Eyprepocnemis plorans* [27], *Locusta migratoria* [28], and *Rhammatocerus brasiliensis* [29], [30]. These markers shed light on the possible origin and evolutionary differentiation of B chromosomes in these taxa [28]–[31]. Although Bs have been frequently investigated, knowledge regarding their origin and specific molecular composition is limited.

*Eyprepocnemis plorans* and *L. migratoria* are the two model species most commonly used to study B chromosome biology in grasshoppers and animals. Over the years, information has been accumulated in these species regarding B chromosome population dynamics, their possible origin, B chromosome gene activity and the interference in the expression of A complement genes due its presence using distinct cytogenetic and molecular approaches [28], [31]–[39]. In contrast, knowledge regarding molecular composition obtained in other grasshopper species such as *D. pratensis* [26], *Podisma kanoi* [40] and *R. brasiliensis* [29], [30] remains limited.

In an attempt to further understand karyotypes and B chromosome composition and evolution in grasshoppers, we analyzed the karyotypic structure and B chromosomes of the South American grasshopper species *Abracris flavolineata* (Acrididae, Ommatolampinae). This species exhibits a karyotype composed of 2n = 23,X0 (males) with a distinct biarmed B chromosome in the population of Rio Claro/SP, Brazil [41]. Specifically, general chromosomal characteristics and B chromosome frequency and structure were studied using classical cytogenetic techniques and mapping of multigene families, telomeric repeats and repeated DNA fraction (*C_0_t*-1 DNA fraction). Our analyses provide new information regarding chromosomal variability in grasshoppers and the specific molecular composition of B chromosomes.

## Materials and Methods

A total of 65 *A. flavolineata* adult individuals, including 38 males and 27 females, were collected in Rio Claro/SP, Brazil with the authorization of ICMBio SISBIO (process number 16009-1). The animals were anesthetized before dissecting testis follicles and gastric caeca. Chromosomes were obtained from male testis follicles and female gastric caeca, which contain mitotic chromosomes, according to the procedure described by Castillo et al. [42]. The tissues used to obtain chromosomes were fixed in modified Carnoy's solution (3∶1, 100% ethanol:glacial acetic acid), and entire animals were stored in 100% ethanol for DNA extraction.

All individuals were studied using conventional staining with 5% Giemsa to estimate B chromosome presence and frequency. The C-banding was obtained as described by Sumner [43], and to identify G+C or A+T rich regions fluorochrome staining (CMA_3_/DA/DAPI) was performed as proposed by Schweizer et al. [44].

Genomic DNA was extracted from the posterior legs of animals with and without one B chromosome using phenol-chlorophorm procedure as proposed by Sambrook and Russel [45]. The DNA was used as template to obtain distinct multigene families such as 5S rDNA, H3 histone and U2 small nuclear RNA (snRNA) genes by polymerase chain reaction (PCR) using primers described by Cabral-de-Mello et al. [46] for 5S rDNA, and by Colgan et al. [47] for H3 histone. Moreover, a specific pair of primers designated by sequences deposited in GenBank as U2F (5′-ATC GCT TCT CGG CCT TAT G–3′) and U2R (5′-TCC CGG CGG TAC TGC AAT A-3′) were used for U2 snDNA amplification. The U1 snDNA sequence was obtained from the *R. brasiliensis* genome using primers described by Cabral-de-Mello et al. [9], and 18S rDNA was obtained from cloned fragments of the *Dichotomius semisquamosus* (GenBank accession number GQ443313.1) genome [46]. All sequences obtained by PCR were sequenced to confirm the sequence of interest, and were deposited in GenBank under the accession numbers KC936996-5S rDNA, KC896792-H3, KC896793-U1 snDNA and KC896794-U2 snDNA. A BLAST search using these sequences confirmed the isolation of the elements that were used as probes. Telomeric probes were obtained by PCR using complementary primers (TTAGG)_5_ and (CCTAA)_5_. Repetitive DNA-enriched samples from individuals with and without one B chromosome were obtained based on the renaturation kinetics of *C_0_t*-1 DNA (DNA enriched for highly and moderately repetitive DNA sequences) according to the protocol described by Zwick et al. [48] with modifications later published [46]. DNA samples (200 µl of 100–500 ng/µl genomic DNA in 0.3 M NaCl) were digested with 0.01 U/µl Deoxyribonuclease I (Sigma) for 1 min and 10 sec to 1 min and 45 sec depending on the sample concentration, and the fragmented DNA was separated by 1% agarose gel electrophoresis. The expected DNA fragments ranged in size from 100 to 1,000 base pairs (bp). DNA fragment samples (50 µl) were denatured at 95°C for 10 min, placed on ice for 10 s and transferred into a 65°C water bath for reannealing for 25 min. Subsequently, the samples were incubated at 37°C for 8 min with 1 U of S1 nuclease to permit the digestion of single-stranded DNA, which was then purified/extracted using a traditional phenol–chlorophorm procedure.

The 5S rDNA, telomeric probes and U1 and U2 snRNA gene probes were labeled through PCR with digoxigenin-11-dUTP (Roche, Mannheim, Germany), and the 18S rDNA, H3 histone gene and *C_0_t*-1 DNA were labeled using biotin-14-dATP through nick translation (Invitrogen, San Diego, CA, USA). Fluorescent *in situ* hybridization (FISH) was performed according to the protocol proposed by Pinkel et al. [49] with modifications described by Cabral-de-Mello et al. [46]. Single or two color FISH were performed with the distinct probes and at least 200 ng of each probe was used. Probes labeled with digoxigenin-11-dUTP were detected using anti-digoxigenin rhodamine (Roche), and probes labeled with biotin-14-dATP were detected using Alexa Fluor 488 (Invitrogen). All preparations were counterstained with 4′,6-diamidino-2-phenylindole (DAPI) and mounted in Vectashield (Vector, Burlingame, CA, USA). Chromosomes and signals were observed using an Olympus microscope BX61 equipped with fluorescence lamp and appropriate filters. Photographs were recorded with a DP70 cooled digital camera. Images were merged and optimized for brightness and contrast with Adobe Photoshop CS2.

For the measurement of relative extension occupied by repetitive DNAs (i.e. C-positive blocks, 18S rDNA, H3 histone and *C_0_t*-1 DNA) the software ImageJ was used. We comparatively analyzed the extension occupied by the C-positive blocks or FISH signals with repetitive DNAs in relation of the whole chromosomal extension in six metaphases. The analyses were performed using mitotic chromosomes from individuals that presented the rDNA distribution showed in the last row of table 1, considering only the chromosomes harboring these clusters.

**Table 1:** Intrapopulational polymorphisms of 18S rDNA in*A. flavolineata.*

Figure	Chromosomes												Total of elements labeled	Frequency observed
	1	2	3	4	5	6	7	8	9	10	11	X		
S1a	p		p	p*	p	p		p#	p	p		p	9	1
S1b	p		p	p*	p	p		p#	p	p			8	1
S1c	p		p		p	p			p	p			6	2
S1d	p	p*	p		p*	p			p	p			7	2
S1e	p	p*	p			p			p	p			6	1
S1f	p		p			p			p	p			5	1
Fig. 2a	p		p		p	p			p				5	1

p: pericentromeric;

* heteromorphic pair;

# tiny signal.

## Results

**Figure 1:**
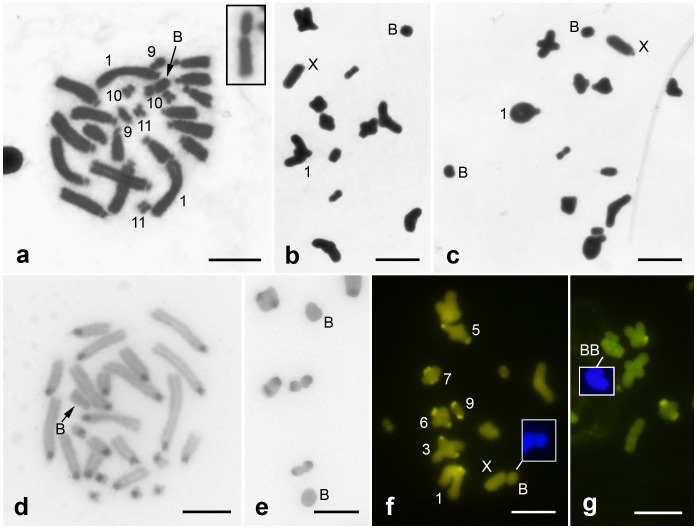
Classical cytogenetic characterization of*Abracris flavolineata* chromosomal complement at mitotic metaphase (a,d) and metaphase I (b,c,e–g) cells. (a–c) conventional staining, (d,e) C-banding and (f,g) CMA_3_ fluorochrome staining in cells with one or two B chromosomes. Insets show the B element obtained from another mitotic metaphase (a), and B chromosomes stained with DAPI (f,g). The B and X chromosomes are indicated in all cells, and (f) chromosomes with CMA_3_-positive blocks are also indicated. The metaphases (e,g) are partial. Bar = 5 µm.

The *A. flavolineata* karyotype is composed of 2n = 23,X0 (males) and 2n = 24,XX (females). Pairs 1–8 and the X are subtelo-acrocentric, pair 9 is submetacentric, and pairs 10 and 11 are metacentric (Figure 1a). This macro chromosomal structure is similar to that previously reported by Cella and Ferreira [41]. Among the 65 animals studied, 30.7% (12 males and 8 females) carried one or two submetacentric B chromosomes (Figure 1a–c). The frequency of B chromosomes was similar in males (31.5%) and females (29.7%). In males, the B chromosome showed mitotic instability in the germ line since primary spermatocytes with 0–1 or 0–2 B elements were observed in eight individuals (Figure 1b,c).

C-positive chromosome regions were concentrated in the pericentromeric region extending to the short chromosomal arms of all chromosomes, while in the B chromosomes no C-positive regions were observed (Figure 1d,e). The heterochromatin blocks of pairs 1, 3, 5, 6, 7 and 9 were G+C-rich, while the other regions were neutral for both fluorochromes (CMA_3_/DAPI) (Figure 1f). DAPI staining did not reveal any positive blocks, as the chromosomes were homogeneously stained (result not shown). No A+T- or G+C-rich blocks were observed in the B chromosomes (Figure 1f,g).

**Figure 2:**
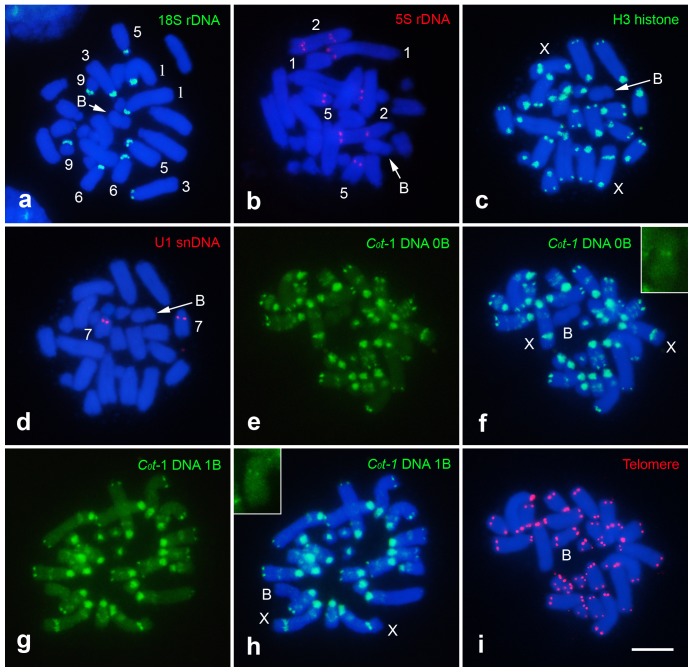
Cytogenetic mapping of repetitive DNAs in gastric caeca female mitotic cells bearing one B chromosome. Each probe used is indicated directly in the images using colors. Insets in (f,h) show the B chromosome with a faint centromeric signal for the *C_0_t*-1 DNA probe. Bar = 5 µm.

In the nine animals analyzed for FISH with the 18S rDNA probe, we observed extensive variation between individuals in the number of chromosomes (5–9) harboring this kind of repetitive DNA, including autosomes and X chromosomes. In the B chromosome no signal for 18S rDNA was observed. All rDNA blocks were located in the short arm of carrier chromosomes (Figure 2a, Table 1, Figure S1), and heteromorphic pairs were observed in some cases (see Figure S1). Table 1 shows that i) rDNA was found in all A chromosomes except 7 and 11, ii) there was no variation in autosomes 1, 3, 6 and 9, and iii) it was polymorphic in the remaining A chromosomes (2, 4, 5, 8, 10 and X). The 5S rDNA location, however, was highly conserved since all individuals carried a distal cluster in the long arm of autosome 1, two interstitial clusters in autosome 2, and one proximal cluster in autosome 5 (Figure 2b).

Remarkably, H3 histone genes were found in all chromosomes, with large pericentromeric clusters in all chromosomes, except the B chromosome. The H3 histone pericentromeric clusters in some chromosomes were extended to the short arms and additional distal clusters in pairs 1–6 and 8, and a large interstitial cluster in the X chromosome was observed (Figure 2c).

U1 snRNA genes were located exclusively in the proximal region of autosome pair 7 (Figure 2d). FISH for highly and moderately repeated DNAs (*C_0_t*-1 DNA), with probes obtained from genomic DNA of 0B and 1B individuals, labeled large proximal regions (including short arms) in all A chromosomes, as well as small terminal blocks in all chromosomes but 7, 9–11, and a large interstitial block in the X chromosome. B chromosomes showed, with both *C_0_t*-1 probes, a very small hybridization signal in the centromere (Figure 2e–h). This signal was not observed in meiotic cells, most likely due to high chromosome condensation, which limits FISH resolution. FISH with the telomeric DNA probe revealed terminal signals in all chromosomes, including the B elements (Figure 2i). A comparative analysis, using mitotic chromosomes, of repetitive DNAs located near to centromeres (heterochromatin, 18S rDNA, H3 histone and *C_0_t*-1 DNA) revealed larger relative region, in size, occupied physically by *C_0_t*-1 DNA in comparison to other repeated DNAs (Figure S2, Table S1). All FISH analyses were also performed in individuals harboring two B chromosomes, and identical results described for individuals with one B were observed (Figure S3).

**Figure 3:**
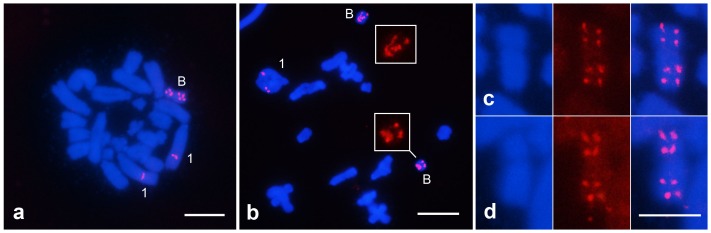
FISH with the U2 snRNA gene probe in individuals with one (a) and two (b) B chromosomes. (a) gastric caeca female mitotic metaphase cell, (b) male metaphase I cell, (c,d) selected B chromosomes showing the symmetrical location of the U2 snDNA clusters in the two arms. Note the double signal in each arm of the B elements. (a,b) Bar = 5 µm, (c,d) Bar = 2.5 µm.

Finally, FISH with the U2 snDNA probe revealed the presence of a single cluster in the A chromosomes, specifically in an interstitial region of the largest autosome. Additionally, in the B chromosome the U2 snDNA presented high copy number with four blocks in this element, two in each arm in a nearly symmetrical location (Figure 3).

## Discussion

### The A complement and dynamics of repetitive DNAs in the*A. flavolineata* genome

The karyotype and heterochromatin distribution observed in *A. flavolineata* is similar to the previous description provided by Cella and Ferreira [41]. These results mirror the common pattern seen in grasshoppers [50], with the exception of the presence of meta- or submetacentric chromosomes (pairs 9–11), which are less common in acridid karyotypes and may be related to pericentric inversions. The absence of interstitial telomeric DNA indicates that either the breakpoints for chromosomal inversions were located outside of telomeric regions or that these repeats were lost after the inversions took place. Alternatively accumulation of repetitive DNAs in short arms could explain the occurrence of meta-/submetacentric chromosomes [41].

The variability observed in base pair composition for C-positive regions may indicate the dynamics related to their specific composition. The *C_0_t*-1 DNA fraction provided additional information concerning repetitive DNAs distribution, revealing that in addition to the high amount of this genome fraction in C-positive regions, repetitive DNAs are also enriched in the terminal and interstitial regions. Additionally, it is noticeable that the regions occupied by *C_0_t*-1 DNA signals were larger in physical size if compared to other repetitive DNAs, including C-positive blocks, H3 histone genes and 18S rDNA, indicating that the pericentromeric regions harbor other repetitive elements. In general, the mapping of *C_0_t*-1 DNA in animals has been a valuable tool in the understanding of repetitive DNA diversification regarding heterochromatin [51], possible sex chromosomes [52] and the origin/evolution of B chromosomes [46], [53] such as the B of *A. flavolineata* (see discussion below).

Chromosomal mapping of the five multigene families in *A. flavolineata* revealed distinct genome dynamics among the sequences studied. This intense difference was observed by contrasting the patterns observed for stable U snRNA genes with only one site, and H3 histone and 18S rRNA genes that are spread and are additionally polymorphic for 18S rDNA. Although the mapping of U1 and U2 has been poorly explored, the cytogenetic mapping of U1 snRNA genes was reported as being highly conserved in cichlid fish and crustaceans, with only one site in the distantly related cichlid fish and more dynamic sites in crustaceans [9], [54], [55]. For U2 snDNA, the occurrence of only one site as observed in *A. flavolineata* was previously described in fish, such as *Halobatrachus didactylus* and *Plectorhinchus mediterraneus*. However, interestingly distinct scenarios were observed in Batrachoididae fish with sites concentrated in one chromosomal pair, sites dispersed in some chromosomes and both organizations in the same genome [56], [57].

The marked genomic dispersal of H3 histone clusters observed in the *A. flavolineata* genome was also reported in the grasshopper *R. brasiliensis* [30], although without the occurrence of terminal and interstitial clusters. This surprising chromosomal distribution in *A. flavolineata* contrasts strongly with the conserved pattern observed in other animals, which possess only a single locus or few loci of histone genes [5], [7], [58]–[60]. Cabrero et al. [5], claimed that, in grasshoppers, the conservation of a number of H3-H4 histone genes dates to the origin of Acrididae, which is reinforced by the analysis of H3 histone clusters in ancient grasshoppers belonging to the Proscopiidae family [60]. The spreading of histone H3 repeats observed in this work suggests greater dynamism of these repeats in grasshoppers. Although deeper analysis is necessary, the role of transposable elements (TE) in histone H3 dispersion must be considered. Furthermore, other mechanisms of repetitive DNA dispersal across the genome may also govern the intense sequence dispersion observed here, such as extrachromosomal circular DNAs (eccDNA), and ectopic recombination, as described for rDNAs [4], [14], [15], [17], [19].

Although less variable than histone H3 genes, 5S rDNA was present at multiple loci, which is a common placement for grasshoppers [8]. Concerning the major rDNA cluster, a more detailed scenario has been described in insects, and high variability has been primarily described at the interspecific level [4], [6], [7], [61]. Variability at the intraspecific level as observed in *A. flavolineata* is less common and was observed in *E. plorans*, Orthoptera [62], in some Scarabaeinae beetles, Coleoptera [7] and in *Triatoma infestans*, Heteroptera [61]. These variations in insects with major rDNA clusters have been frequently attributed to ectopic recombination, transpositions, translocations, structural rearrangements and gene conversion followed by amplifications [4], [6], [7], [61].

### The B chromosome

The presence of a B chromosome in *A. flavolineata* was described for the first time by Cella and Ferreira [41] in the same population analyzed in this study, but without frequency description. The main difference observed in this work with the previous description is the morphology of the B element, which was previously classified as metacentric and in our analysis, it was submetacentric.

The absence of C-positive regions in the B chromosome initially indicated the possible low quantity of repeated DNAs. Additionally, the use of the repetitive DNA fraction (*C_0_t*-1 DNA) obtained from genomes with or without one B chromosome as probes indicated a possible low copy number of B-specific repetitive DNAs, such as satellites, or that this element possesses a high number of different repetitive sequences not represented in the *C_0_t*-1 DNA fraction. Our results indicate that in addition to the non-accumulation of some repetitive DNAs in the B chromosome, this element does not share the general pool of repetitive DNAs with the A genome, except for less repeated elements such as U2 snRNA, and some transposable elements (unpublished results), possibly not represented in the *C_0_t*-1 DNA fraction isolated, besides by the occurrence of a faint and punctual signal in the B centromere. This pattern suggests the non-homogenization of the A complement and B chromosomes in *A. flavolineata* and it could indicates a recent origin of this chromosome due to the non-accumulation of repetitive DNAs. This non-accumulation of repetitive DNAs in the B chromosome is contrary to a common pattern of B evolution, with accumulation of repetitive DNAs in this element as observed in some species [21], [24], [25]. Similarly, a lack of DNA identity between A and B chromosomes was described in the fish *Prochilodus lineatus* [63], which contrasts with some common cases of A and B chromosome DNA sharing in animals such as in *E. plorans* [33], *L. migratoria* [34], *Podisma kanoi* [40], *P. sapporensis* [64], *Dichotomius geminatus* [46], *Astyanax scabripinis* [53], *Vulpes vulpes* [65] and *Apodemus peninsulae* [66].

Chromosomal mapping of multigene families provided interesting information regarding the genome dynamics of *A. flavolineata* and the possible autosomal B origin/diversification in this species. The remarkable presence of U2 snRNA genes in the B element is reasonable evidence for its ancestry to the autosomal pair 1, the unique element that harbors a large cluster of this sequence observed by FISH. Autosomal origins for B chromosomes in grasshoppers were also proposed for example in *Dichroplus pratensis* [26], *R. brasiliensis*, *Xyleus discoideus angulatus* [29] and *L. migratoria* [28] using repetitive DNA mapping.

The presence of other multigene families were described in distinct B chromosomes in grasshoppers, such as 45S rDNA [26], [27], 5S rDNA [30], [31] and H3/H4 histone genes [28], [30]; however, the case of *A. flavolineata* is the first demonstration of the occurrence of the U2 snRNA gene in B chromosomes among eukaryotes. In contrast to U2 snDNA, the other multigene families used as probes in *A. flavolineata*, i.e., 18S rRNA and 5S rRNA, H3 histone and U1 snRNA genes, revealed the absence of signals in the B chromosome. The specific cases of H3 histone and 18S rRNA genes are interesting, although highly variable in the number and position for 18S rDNA, and the intense dispersion of H3 histone these sequences were not transposed to the B chromosomes. The dispersion of H3 histone genes could have occurred after the origin of the B chromosome in the genome of *A. flavolineata*. These aspects reinforce the hypothesis of B origin from pair 1 (with U2 snDNA), which is apparently one sequence without high transposition dynamics in the *A. flavolineata* genome, considering the occurrence of a single locus. We could not, however, definitively rule out the possibility of a transposition of U2 snDNA sequences to the B element after its origin followed by amplification.

Additionally, the similar distribution of U2 snDNA clusters in the two arms of the B chromosome led us to hypothesize the origin of this chromosome based on isochromosomes, followed by the enlargement of one chromosomal arm or the occurrence of a pericentric inversion, which causes the difference in the size of the arms. The origin of the B chromosome through isochromosomes was observed, for example in the fish *Astyanax scabripinis* [67] and *Prochilodus lineatus* [68], other grasshoppers [69]–[73], and plants such as *Brachycome dichromosomatica* [25] and rye *Secale cereale* [74].

The classical cytogenetic methods and the mapping of repeated DNAs in individuals harboring two B chromosomes indicated similarity between these elements, suggesting that only one type of B chromosome is present in the population studied. This observation contrasts with the high variability of the molecular composition or the sequence distribution in B elements, as observed in *E. plorans* [31], [75] and *P. lineatus* [68]. The variability of B elements has been attributed to the differential amplification/deletion of DNA sequences in addition to the involvement of translocations/transpositions of sequences from the A genome and organellar DNA [20], [25], [53], [76], [77]. Although the B chromosome of *A. flavolineata* did not present variability, we could not rule out the occurrence of the mechanism cited above, intrinsic for B evolution, and certainly the analysis of other sequences will shed light on this issue.

The data presented provide new information regarding chromosomal evolution of repetitive sequences in grasshoppers and revealed intense dynamics for 18S rDNA and H3 histone genes in *A. flavolineata*, indicating, in the case of H3 histone genes, that this sequence could be more dynamic than previously reported in Acrididae grasshopper genomes [5]. U2 snDNA can be used as an interesting marker to investigate B chromosome origin/evolution by providing new information concerning B chromosome composition in eukaryotes. The use of this marker in the case of *A. flavolineata* highlighted the autosomal origin and conservation of the B element, at least in the Rio Claro/SP population. Finally, the analysis of other populations conducted using the U2 snRNA gene will provide information regarding the origin and evolution of this polymorphism in this species, as well as the use of other chromosomal markers through FISH.

## Supporting Information

Figure S1 **Meiotic cells from distinct individuals of** ***A. flavolineata*** **showing the variable patterns of 18S rDNA distribution.** Autosomal bivalents were numbered in order of decreasing size. Bar = 5 µm.
(PDF)

Figure S2 **Selected mitotic chromosomes of** ***A. flavolineata*** **after C-banding treatment and FISH with 18S rDNA, H3 histone and** ***C_0_t*** **-1 DNA as probes.** Note the occurrence of large *C_0_t*-1 DNA blocks. Blue = DAPI, Green = signals.
(PDF)

Figure S3 **Partial metaphases I of** ***A. flavolineata*** **individuals harboring two B chromosomes.** The probes used are indicated in colors directly in each cell. Bar = 5 µm.
(PDF)

Table S1 **Relative length occupied by repetitive DNAs in chromosomes 1, 3, 5, 6 and 9 of** ***A. flavolineata*** **.** Note that in all chromosomes the region occupied by *C_0_t*-1 DNA is larger than for other repetitive DNAs.
(PDF)
