# Supplementary figures and images for: Chromosomal Mapping of Repetitive DNAs in the Grasshopper Abracris flavolineata Reveal Possible Ancestry of the B Chromosome and H3 Histone Spreading
Source: PLoS One. 2013 Jun 27;8(6):e66532. doi: 10.1371/journal.pone.0066532 (PMC3694960; doi:10.1371/journal.pone.0066532)

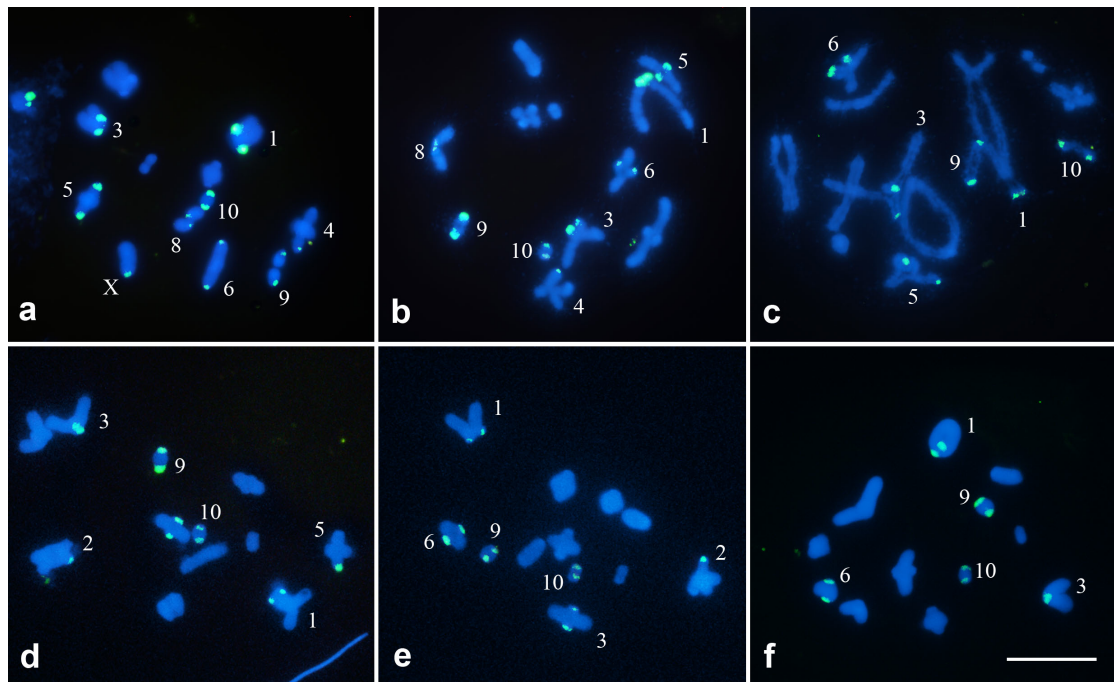

Supplement: Figure S1 — Meiotic cells from distinct individuals of A. flavolineata showing the variable patterns of 18S rDNA distribution. Autosomal bivalents were numbered in order of decreasing size. Bar = 5 µm. (PDF) [file pone.0066532.s001.pdf]

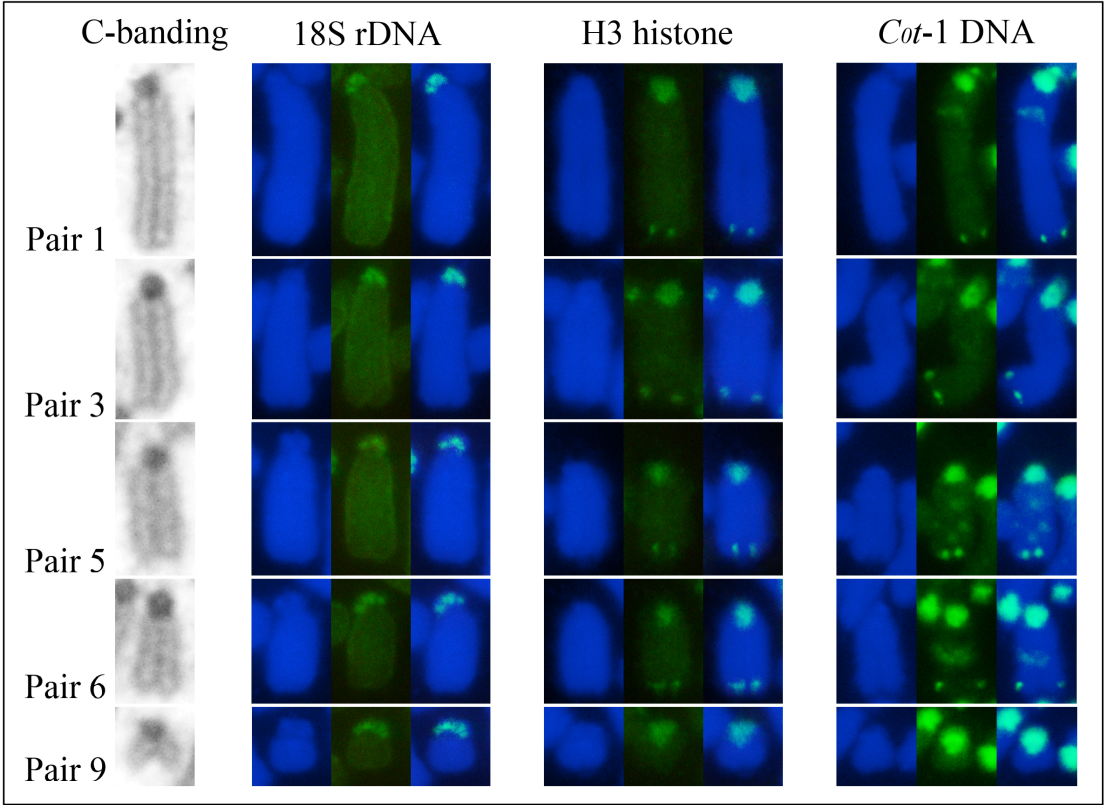

Supplement: Figure S2 — Selected mitotic chromosomes of A. flavolineata after C-banding treatment and FISH with 18S rDNA, H3 histone and C0t -1 DNA as probes. Note the occurrence of large C0t-1 DNA blocks. Blue = DAPI, Green = signals. (PDF) [file pone.0066532.s002.pdf]

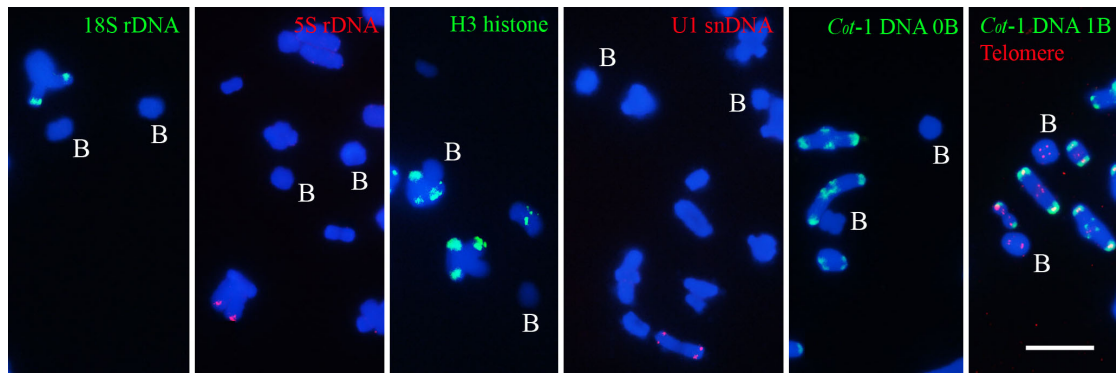

Supplement: Figure S3 — Partial metaphases I of A. flavolineata individuals harboring two B chromosomes. The probes used are indicated in colors directly in each cell. Bar = 5 µm. (PDF) [file pone.0066532.s003.pdf]
