# Supplementary material for: Chromosomal Mapping of Repetitive DNAs in the Grasshopper Abracris flavolineata Reveal Possible Ancestry of the B Chromosome and H3 Histone Spreading
Source: PLoS One. 2013 Jun 27;8(6):e66532. doi: 10.1371/journal.pone.0066532 (PMC3694960; doi:10.1371/journal.pone.0066532)
Supplement: Table S1 — Relative length occupied by repetitive DNAs in chromosomes 1, 3, 5, 6 and 9 of A. flavolineata . Note that in all chromosomes the region occupied by C0t-1 DNA is larger than for other repetitive DNAs. (PDF) [file pone.0066532.s004.pdf]

| **Chromosome pair** | **C-positive blocks** | **18S rDNA** | **H3 histone** | ***$C_0t$*-1 DNA** |
|---|---|---|---|---|
| 1 | 11,40% | 7,40% | 11,83% | 14,00% |
| 3 | 18,40% | 8,96% | 17,13% | 23,57% |
| 5 | 19,4% | 7,10% | 17,85% | 21,00% |
| 6 | 23,80% | 10,63% | 22,58% | 26,85% |
| 9 | 30,70% | 16,90% | 37,50% | 58,60% |
